# Development of Wind Speed Retrieval from Cross-Polarization Chinese Gaofen-3 Synthetic Aperture Radar in Typhoons

**DOI:** 10.3390/s18020412

**Published:** 2018-01-31

**Authors:** Weizeng Shao, Xinzhe Yuan, Yexin Sheng, Jian Sun, Wei Zhou, Qingjun Zhang

**Affiliations:** 1 Marine Science and Technology College, Zhejiang Ocean University, Zhoushan 316022, China; shaoweizeng@zjou.edu.cn (W.S.); shengyexin@hotmail.com (Y.S.); 2 National Satellite Ocean Application Service, State Oceanic Administration, Beijing 100081, China; 3 Physical Oceanography Laboratory/CIMST, Ocean University of China and Qingdao National Laboratory for Marine Science and Technology, Qingdao 266100, China; sunjian77@ouc.edu.cn; 4 South China Sea Institute of Oceanology, Chinese Academy of Sciences, Guangzhou 510301, China; zhouwei@scsio.ac.cn; 5 Beijing Institute of Spacecraft System Engineering, Beijing 100076, China; ztzhangqj@163.com

**Keywords:** wind speed, typhoon, cross-polarization, Gaofen-3 SAR

## Abstract

The purpose of our work is to determine the feasibility and effectiveness of retrieving sea surface wind speeds from C-band cross-polarization (herein vertical-horizontal, VH) Chinese Gaofen-3 (GF-3) SAR images in typhoons. In this study, we have collected three GF-3 SAR images acquired in Global Observation (GLO) and Wide ScanSAR (WSC) mode during the summer of 2017 from the China Sea, which includes the typhoons Noru, Doksuri and Talim. These images were collocated with wind simulations at 0.12° grids from a numeric model, called the Regional Assimilation and Prediction System-Typhoon model (GRAPES-TYM). Recent research shows that GRAPES-TYM has a good performance for typhoon simulation in the China Sea. Based on the dataset, the dependence of wind speed and of radar incidence angle on normalized radar cross (NRCS) of VH-polarization GF-3 SAR have been investigated, after which an empirical algorithm for wind speed retrieval from VH-polarization GF-3 SAR was tuned. An additional four VH-polarization GF-3 SAR images in three typhoons, Noru, Hato and Talim, were investigated in order to validate the proposed algorithm. SAR-derived winds were compared with measurements from Windsat winds at 0.25° grids with wind speeds up to 40 m/s, showing a 5.5 m/s root mean square error (RMSE) of wind speed and an improved RMSE of 5.1 m/s wind speed was achieved compared with the retrieval results validated against GRAPES-TYM winds. It is concluded that the proposed algorithm is a promising potential technique for strong wind retrieval from cross-polarization GF-3 SAR images without encountering a signal saturation problem.

## 1. Introduction

Synthetic aperture radar (SAR) operates in all-weather conditions and has the capability to detect sea surface winds, especially under extreme wind conditions [1,2]. Currently, SAR data is available from C-band (5.3 GHz) Radarsat-2 (R-2) and Sentinel-1 (S-1); X-band (9.8 GHz) TerraSAR-X/TanDEM-X, and Cosmo-SkyMed; and L-band (1.2 GHz) ALOS-2 satellite. The new generation C-band Gaofen-3 (GF-3) SAR was launched by the China Academy of Space Technology (CAST) in 2016 with a wide spatial swath coverage, i.e., more than 500 km swath for Global Observation (GLO) mode and Wide ScanSAR (WSC) mode. So far, GF-3 SAR supports operations in alternative dual-polarization, i.e., vertical-vertical (VV) with vertical-horizontal (VH) or horizontal-horizontal (HH) with horizontal-vertical (HV). Our institutions are the primary participants involved in the development of marine applications using GF-3 SAR data, in particular, for wind and wave monitoring.

Wind retrieval from C-band co-polarization (VV and HH polarization) SAR is a now a mature technique. Geophysical Model Functions (GMFs) for co-polarization C-band SAR wind retrieval CMODs have been widely used over the last few decades, i.e., CMOD4 [3], CMOD-IFR2 [4], CMOD5 [5], CMOD5N for neutral winds [6] and the new GMF C-SARMOD [7]. The C-band GMFs describe a complicated relationship between the normalized radar cross section (NRCS) of co-polarization SAR and wind vectors at 10 m above sea surface. CMODs have been successfully applied for wind retrieval from SAR at C-band and well validated at wind speed ranges from 0–20 m/s [8,9,10,11]. In particular, wind speeds retrieved from a large number of VV and HH polarization S-1 SAR images have been recently validated against the products from the Advanced Scatterometer (ASCAT) on board satellite Metop–A and –B [12]. Results indicate that the CMOD5N and CMOD4 plus polarization ratio (PR) models proposed in [13] are the best options for wind retrieval in VV and HH polarization, respectively. However, because two unknown variables exist in these C-band GMFs, i.e., wind speed and wind direction, it is impossible to derive the wind vector by inverting the individual C-band GMF without any prior information.

It was found that spatial patterns visible on SAR imagery, known as “wind streaks” [14], form parallel to the wind direction. A local gradient (LG) method can be used for retrieving wind directions automatically, as wind direction is considered as be orthogonal to the gradient direction of the local SAR intensity image [15,16]. Unfortunately, SAR-derived wind directions have a 180° ambiguity. In order to remove that ambiguity, an independent source is still required. In practice, the application of C-band GMF directly employs external information on wind direction at low spatial and temporal resolution, i.e., numeric prediction and observation derived from a scatterometer. However, the spatial resolution of external wind direction is coarse, which is a mismatch with SAR imagery, especially in coastal regions. The sensitivity of SAR-wind speed on NRCS of co-polarization SAR reduces, due to the saturation of the backscattering signal under strong wind conditions (probably at wind speeds greater than 25 m/s) [17,18], causing a root mean square error (RMSE) 6.2–6.5 m/s of wind speed for VV-polarization [19]. This is considered to be the one of the major weaknesses for wind retrieval from SAR when using the traditional C-band co-polarization GMF method.

Interestingly, the benefits of cross-polarization (VH and HV polarization) SAR signals have been studied over the past few years. Recent research has revealed that the NRCS of cross-polarization SAR in units of dB has a strong linear relationship with wind speed, while cross-polarization NRCS is less sensitive to wind direction [20]. Several studies have focused on retrieving wind speeds from C-band cross-polarization R-2 SAR [21,22,23,24,25] and S-1 SAR [26], showing that the retrieved wind speeds have a good agreement with moored measurements. It also has been found that cross-polarization SAR radar backscattering from the ocean surface enhances sensitivity to signal saturation [27,28,29,30,31,32], especially under strong wind conditions (probably for winds greater than 20 m/s). A comparison with stepped frequency microwave radiometer (SFMR) wind speeds for some cases in hurricanes has shown strong winds with speeds up to around 55 m/s with a ±5 m/s deviation [21], due to the fact the cross-polarization does not saturate as easily as the co-polarization signal. These apparent advantages bring the expectation of incorporating a VH channel into the next generation of scatterometer designs [33,34].

In this study, the wind fields from the Global and Regional Assimilation and Prediction System—Typhoon model (GRAPES-TYM) at 0.12° grids, which is used for typhoon simulation in the China Sea [35], have been collocated with the VH-polarization GF-3 SAR images taken of typhoons in the China Sea. Through the dataset, the dependence of wind speed and radar incidence angle on the NRCS of VH-polarization GF-3 SAR was investigated. Then we tuned an empirical algorithm for wind speed retrieval from VH-polarization GF-3 SAR image in strong winds ranging from speeds up to 40 m/s. 

We organize the remainder of this paper as follows: Section 2 introduces the datasets used in our study, i.e., GF-3 SAR images in VH-polarization, Windsat polarimetric radiometer at 0.25° grids and wind simulation from numeric GRAPES-TYM. Then an empirical wind retrieval algorithm for VH-polarization GF-3 SAR is tuned in Section 3. The validation of retrieval wind speeds from the VH-polarization GF-3 SAR images is presented in Section 4 and the discussion is presented in Section 5. Section 6 gives the conclusions and summary.

## 2. Description of Dataset

In total, seven VH-polarization GF-3 SAR images, which were acquired in GLO and WSC mode, were collected during the summer of 2017 in the China Sea. These VH-polarization GF-3 SAR images were processed as Level-1B (L-1B) products and were taken under typhoon conditions. They have the standard pixel size of about 100~500 m for range and azimuth direction. A GF-3 SAR image in GLO mode and WSC mode is comprised of several single radar beams. The details of the six collected GF-3 SAR images and information about the corresponding typhoons is listed in Table A1 of the Appendix A, in which the typhoon information has been bilinearly interpolated to the SAR imaging time. We use the following equation for calculating the NRCS of cross-polarization GF-3 SAR acquired in L-1B mode:

(1) $$\sigma^{0}={\mathrm{DN}}^{2}{\left(\frac{M}{65535}\right)}^{2}-N$$

where in σ^0^ is the NRCS in units of dB, DN is SAR-measured intensity, M is the external calibration factor and N is the offset constant stored in the annotation file.

**Figure 1:**
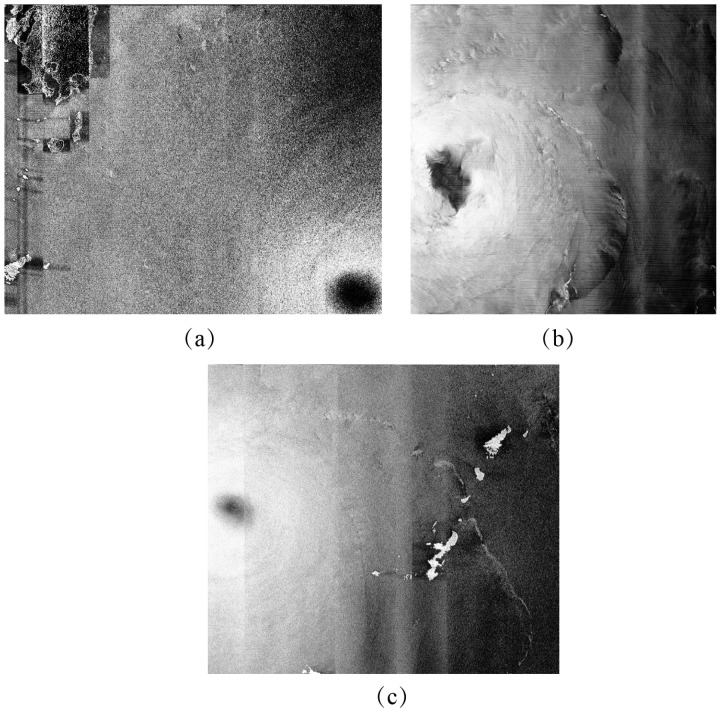
VH-polarization GF-3 SAR images taken during three typhoons. (**a**) The image from typhoon Noru acquired in Global Observation (GLO) mode on 2 August 2017 at 21:09 UTC. (**b**) The image from typhoon Doksuri acquired in Wide ScanSAR (WSC) mode on 13 September 2017 at 22:14 UTC. (**c**) The image from typhoon Talim acquired in GLO mode on 14 September 2017 at 21:29 UTC.

In the data collection, there are three VH-polarization GF-3 SAR images, which were taken during the periods of typhoons Noru, Hato and Doksuri. Figure 1 shows three quick-look images of this. Due to the lower signal-to-noise ratio of dual-polarization, GF-3 SAR suffers from crosstalk among the different polarization channels. The effect of instrumental noise causes the apparently lighter near the edge of different signature beams of GF-3 SAR, as aligned vertically shown in Figure 1. This is assumed to reduce the instrumental noise for wind retrieval. A technique is proposed in [23] of using the information of referred noise, called the ‘de-noised’ procedure. Unfortunately, the instrumental noise information for GF-3 SAR has yet to be stored in the annotation file. Therefore, it is difficult to effectively deal with the instrumental noise in the VH-polarization channel in this study. 

**Figure 2:**
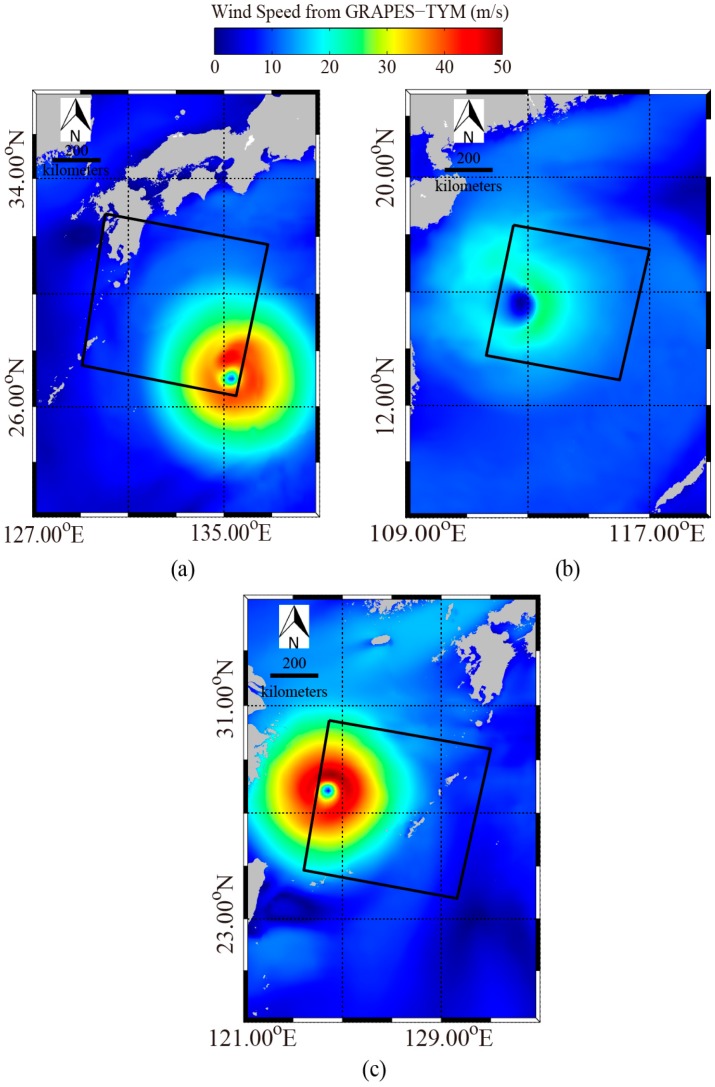
Global and Regional Assimilation and Prediction System—Typhoon model (GRAPES-TYM) wind maps, in which rectangles represent the coverage of the corresponding three VH-polarization GF-3 SAR images in Figure 1. (**a**) GRAPES-TYM wind maps from typhoon Noru on 2 August 2017 at 21:00 UTC. (**b**) GRAPES-TYM wind maps from typhoon Doksuri on 13 September 2017 at 22:00 UTC. (**c**) GRAPES-TYM winds maps from typhoon Talim on 14 September 2017 at 21:00 UTC.

The Windsat polarimetric radiometer was developed by the Naval Research Laboratory (NRL) and has the capability to measure all-weather winds from space with swath more than 350 km. A comparison between Windsat winds at 10 m height above sea surface and SFMR observations shows that Windsat winds do not encounter saturation problems in strong winds ranging from 20 to 40 m/s [36]. In this study, we collected measurements from Windsat at 0.25° grids. However, we found there were only a few Windsat grids in the coverage of the three images in Figure 1. Therefore, GRAPES-TYM wind fields at 0.12° grids were used here and were collocated with those three GF-3 SAR images. The GRAPES-TYM wind maps are shown in Figure 2, in which the rectangles represent the spatial coverage of the three VH-polarization GF-3 SAR images in Figure 1. The time difference between them is within 30 min. It was proved in [35] that GRAPES-TYM wind fields from all the typhoon cases in the northwest Pacific Ocean and South China Sea in 2013 had good performances with observations. Moreover, the GRAPES-TYM winds map information, i.e., locations of typhoon eye (TE) and maximum wind speeds, is consistent with the data listed in Table A1. The matchup dataset has a large number of samples with wind speeds up to 40 m/s, which is suitable for developing a high-wind retrieval algorithm. 

**Figure 3:**
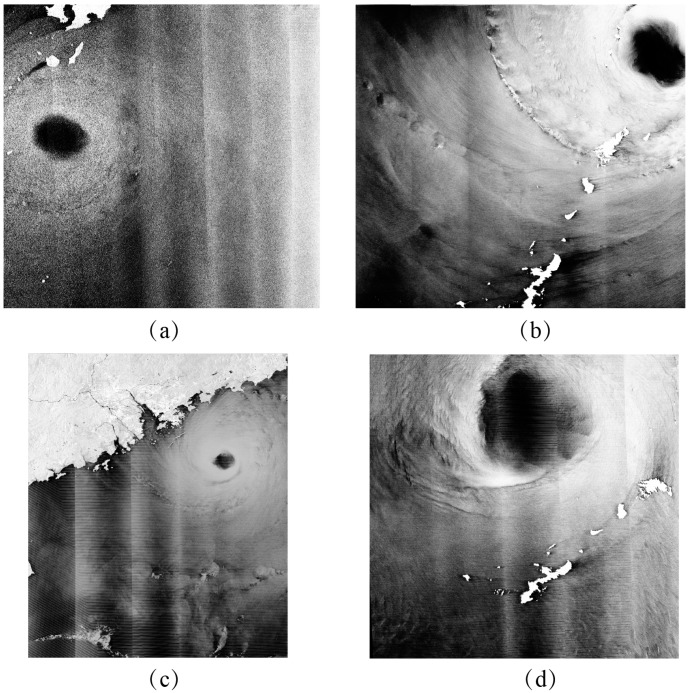
Three VH-polarization GF-3 SAR images taken during the period of three typhoons. (**a**) The image from typhoon Noru acquired in GLO mode on 4 August 2017 at 09:12 UTC. (**b**) The image from typhoon Noru acquired in WSC mode on 4 August 2017 at 21:26 UTC. (**c**) The image from typhoon Hato acquired in WSC mode on 22 August 2017 at 22:23 UTC. (**d**) The image from typhoon Talim acquired in WSC mode on 16 September 2017 at 09:34 UTC.

**Figure 4:**
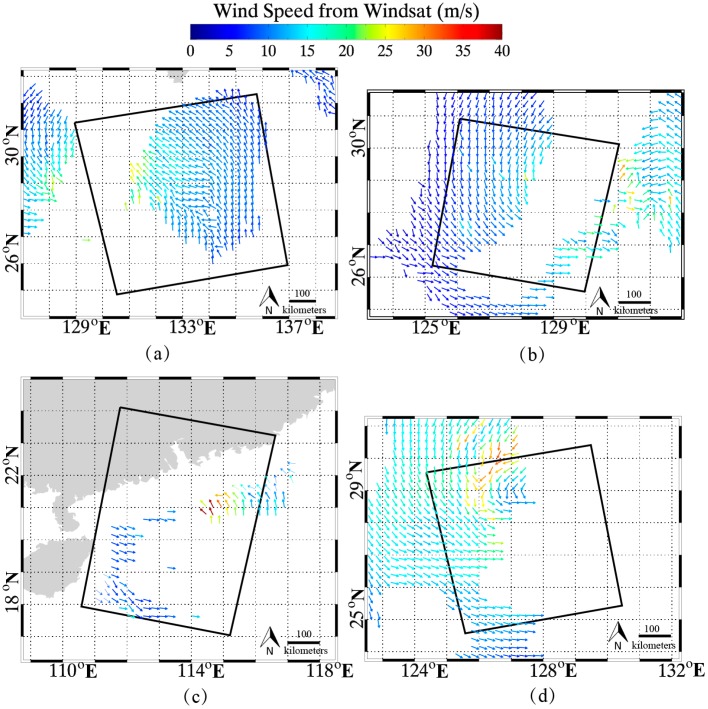
Windsat wind maps corresponding to the four VH-polarization GF-3 SAR images in Figure 2, in which rectangles represent the coverage of the images. (**a**) Windsat wind map from typhoon Noru on 4 August 2017 at 09:18 UTC. (**b**) Windsat wind map from typhoon Noru on 4 August 2017 at 21:18 UTC. (**c**) Windsat wind map from typhoon Hato on 22 August 2017 at 22:30 UTC. (**d**) The Windsat wind map from typhoon Talim on 16 September 2017 at 09:36 UTC.

As well as the three cases, the proposed algorithm has been implemented for the other four VH-polarization GF-3 SAR images during the periods of typhoons Noru, Hato and Talim. The quick-look images of these four GF-3 SAR images and the corresponding Windsat wind maps are shown in Figure 3 and Figure 4, respectively. It can be clearly seen that there are some measurements in the coverage of these four images with wind speeds up to 40 m/s, noting that, the time difference between Windsat winds and the collected four SAR imaging times is within 15 min. Thus, the retrieval wind speeds can be directly validated against Windsat gridded wind speeds. 

## 3. Development of a Wind Retrieval Algorithm for VH-Polarization GF-3 SAR

Recent research has revealed that the NRCS of cross-polarization SAR is independent of wind direction [19,20,21,22]. In this section, the dependence of sea surface wind speed and of radar incidence angle on NRCS of VH-polarization GF-3 SAR are investigated. Based on the findings, we developed an empirical wind retrieval algorithm for VH-polarization GF-3 SAR.

### 3.1. Dependence on VH-Polarization GF-3 SAR NRCS

During the process, each whole SAR image was divided into a number of sub-scenes from squared image blocks with a spatial coverage of about 8 km × 8 km. The sub-scene, covering the GRAPES-TYM winds fields at 0.12° grids, was chosen. Therefore, GRAPES-TYM wind fields at 10 m height above sea surface were matched up with the NRCSs and radar incidence angles from the three VH-polarization GF-3 SAR images in typhoons Noru, Doksuri and Talim. The number of co-locations were more than five thousand samples in about 10% of the total extracted sub-scenes. The collocated dataset was used for studying the dependence of winds and of radar incidence angle on NRCS of VH-polarization GF-3 SAR.

**Figure 5:**
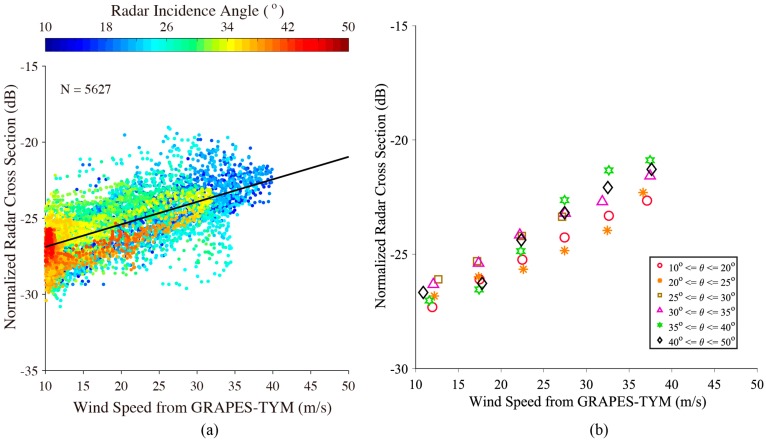
(**a**) The relationship between VH-polarization GF-3 NRCS and wind speed, in which the black line represents the linear fitting result. (**b**) Average NRCS of VH-polarization Gaofen-3 SAR versus wind speed between 10 m/s and 40 m/s for a 5 m/s bin at various incidence angle ranges.

In order to make a reasonable analysis, sub-scenes with winds smaller than 10 m/s and non-homogeneous sub-scenes due to heavy rainfall, where the ratio of image variance and squared image mean value was greater than 1.05 [37,38], were excluded in this study. Figure 5a shows the NRCS of VH-polarization GF-3 SAR versus wind speeds from GRAPES-TYM, in which the color represents the radar incidence angle. Figure 5b shows the average NRCS of VH-polarization Gaofen-3 SAR versus wind speeds between 10 m/s and 40 m/s for a 5 m/s bin at various incidence angle ranges. In general, it is found that the NRCS of VH-polarization GF-3 SAR linearly enhances with the growth of wind speed. This finding is consistent with the conclusions of several previous studies [18,20,31]. It is also shown that the slop between NRCS of VH-polarization GF-3 SAR is almost unique for various radar incidence angles at moderate to strong winds. The range of the reference GRAPES-TYM wind speed is up to 40 m/s and no indication of the signal saturation problem was encountered in the application of traditional wind retrieval algorithms for co-polarization C-band SAR. The black line in Figure 5a represents the linear fitting result between VH-polarization GF-3 NRCS and wind speed.

**Figure 6:**
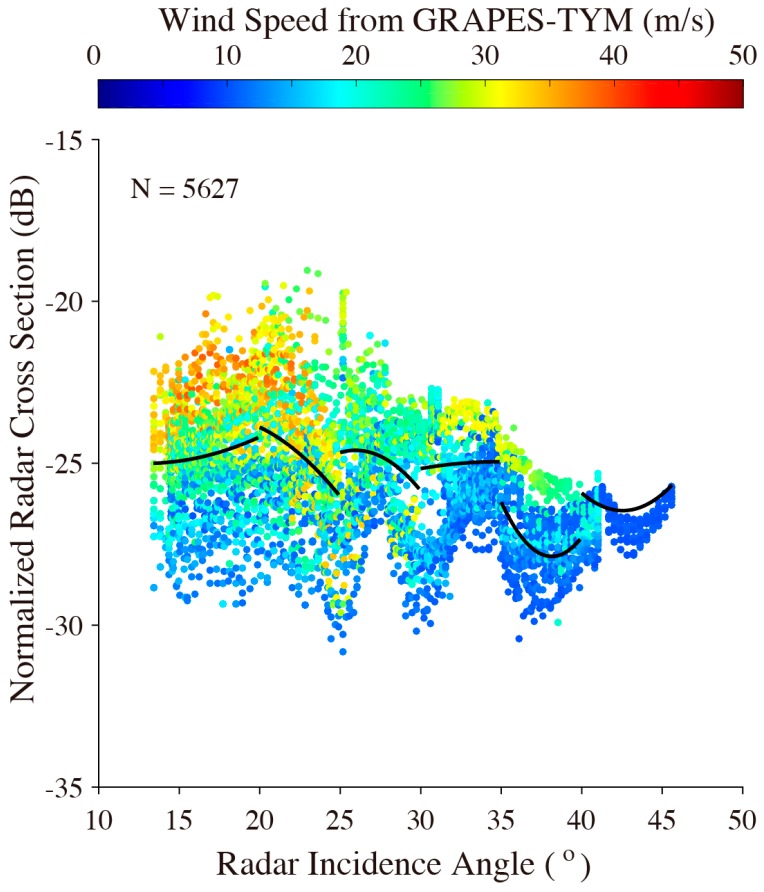
NRCS of entire VH-polarization GF-3 SAR image versus collocated radar incidence angle, in which the black lines represent the fitting results between VH-polarization GF-3 NRCS and radar incidence angle.

Figure 6 shows the NRCS of the entire VH-polarization GF-3 SAR image versus collocated radar incidence angle, in which the color represents the wind speed. A fluctuating relationship can be observed between the NRCS of VH-polarization GF-3 SAR and the radar incidence angle, which is similar to the analyzed results in [26]. The black lines in Figure 6 also represent the fitting results between the VH-polarization GF-3 NRCS and the radar incidence angle.

### 3.2. Tuning the Empirical Algorithm through the Collocated Dataset

Recently, some studies have made great efforts to retrieve winds from cross-polarization R-2 SAR images [19,20,21,22,23,24,25,32]. To date, several cross-polarization wind retrieval models have been exploited. A C-band cross-polarized ocean surface wind retrieval model for dual-polarization SAR (C-2POD) model has been widely used for strong wind retrieval from R-2 SAR images that only include the wind speed term. The C-2POD model takes the general formulation:

σ^0^ = aU_10_ + b [dB] (2)

where in σ^0^ is the VH-polarization NRCS in units of dB, U_10_ is the wind speed at 10 m height above sea surface, a and b are the constants determined for different R-2 mode data, i.e., fine quad-polarization [19,20,21] and dual-polarization ScanSAR mode [22,23,32]. 

In fact, the dependence of sea surface wind speed on NRCS is true for C-band R-2 and GF-3 SAR. However, based on the analysis above, it was found that VH-polarization NRCS of GF-3 SAR fluctuate over the radar incidence angle. For convenient application, we have made an attempt to directly retrieve winds from VH-polarization NRCS of GF-3 SAR at various radar incidence angle ranges. Referring to recent achievements of wind retrieval for cross-polarization S-1 SAR [26], an empirical algorithm is now proposed, including both terms of wind speed and radar incidence angle, and is described as follows:

σ^0^ = f_1_(1 + α‖f_2_‖) + β [dB] (3)

in which, f_1_ is the function of VH-polarization NRCS taking a similar formulation to Equation (3):

(4) $$f_{1}={\mathrm{AU}}_{10}+B$$

f_2_ is the function of radar incidence angle normalized into [−1,1] for various radar incidence angle ranges as GF-3 SAR has a different performance of instrumental noise for each radar beam:

f_2_ = C_1_θ^2^ + C_2_θ + C_3_ (5)

and the coefficients α, β, A, B and matrix C are the tuned constants, as shown in Table 1. The categories of θ are ranged as [10°~20°], from 20° to 40° for a 5° bin and [40°~50°].

**Figure 7:**
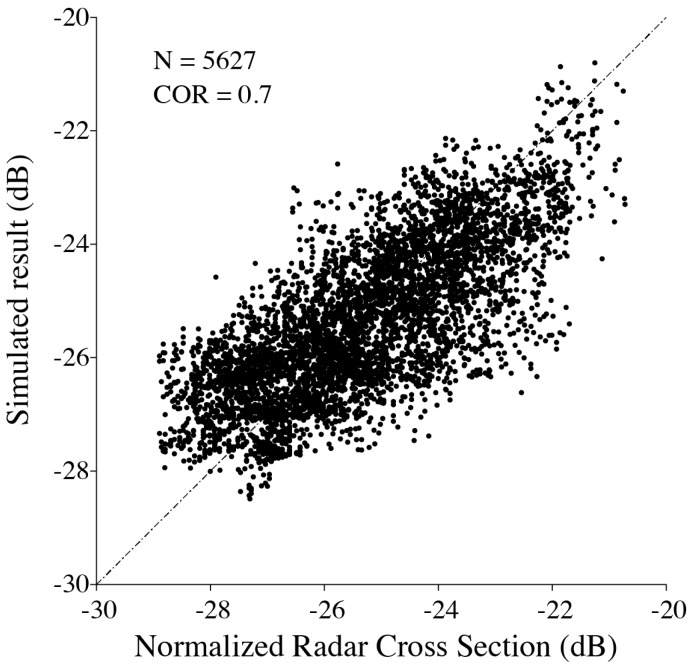
Comparison between observed NRCS of VH-polarization GF-3 SAR and simulated results using the empirical algorithm.

We collected many matchups from the three VH-polarization GF-3 SAR images from the typhoons. As mentioned above, the matchups with wind speeds smaller than 10 m/s are excluded in the tuning process in order to give reasonable fitted results using the least-squares method. Figure 7 shows that the correlation (COR) between the observed NRCS and the simulated values is 0.7, indicating the proposed empirical algorithm is suitable for typhoon wind retrieval. 

## 4. Validation

In addition to the three images used for tuning the algorithm, there are four VH-polarization GF-3 SAR images taken from typhoons Noru, Hato and Talim. The retrieval results are compared with measurements from the Windsat polarimetric radiometer at 0.25° grids, as there are more than four hundred matchups in the collocated dataset. 

**Figure 8:**
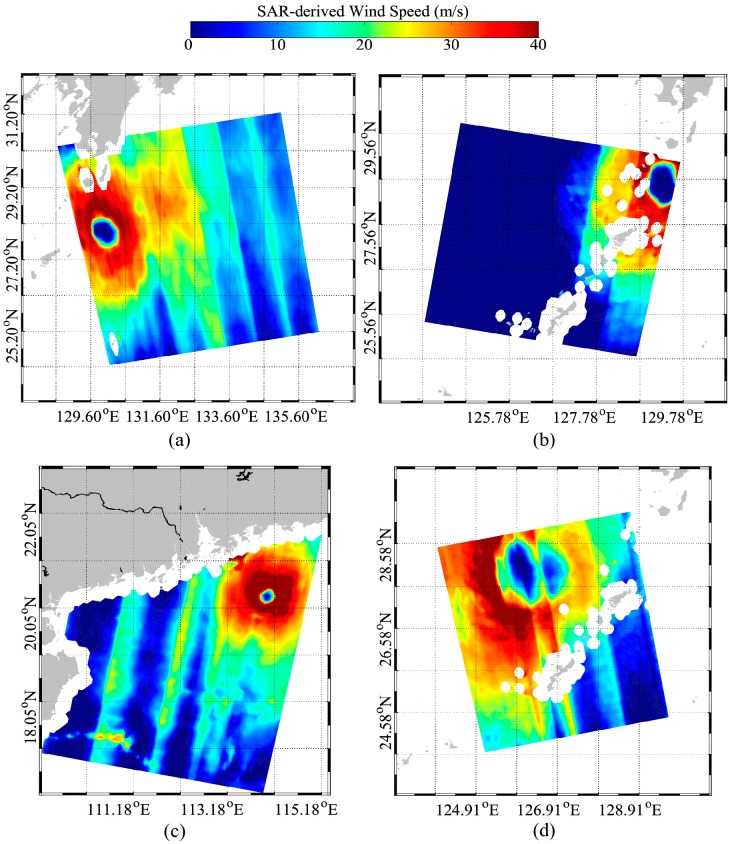
The retrieved wind maps of the four S-1 images. (**a**) Lionrock acquired on 29 August at 08:32 UTC. (**b**) Lester acquired on 31 August at 03:15 UTC. (**c**) Gaston acquired on 1 September at 20:30 UTC. (**d**) Lester acquired on 4 September at 16:31 UTC.

Figure 8 shows the retrieved wind maps corresponding to the four GF-3 images in Figure 3. It is necessary to establish that retrieved winds with wind speeds smaller than 10 m/s are not reliable. Although the Windsat wind speed is up to 40 m/s, the retrieved winds do not encounter the saturation problem as found in the application of C-band co-polarization GMFs. Discontinuities exist in the retrieved winds maps because the GF-3 SAR image acquired in GLO and WSC mode is comprised of several radar beams. In particular, the retrieved TE of typhoon Lester shown in Figure 8d is distorted, because of the instrumental noise of the radar beam. For this reason, the retrieved wind speed is significantly different around the edge of each radar beam than in other regions.

**Figure 9:**
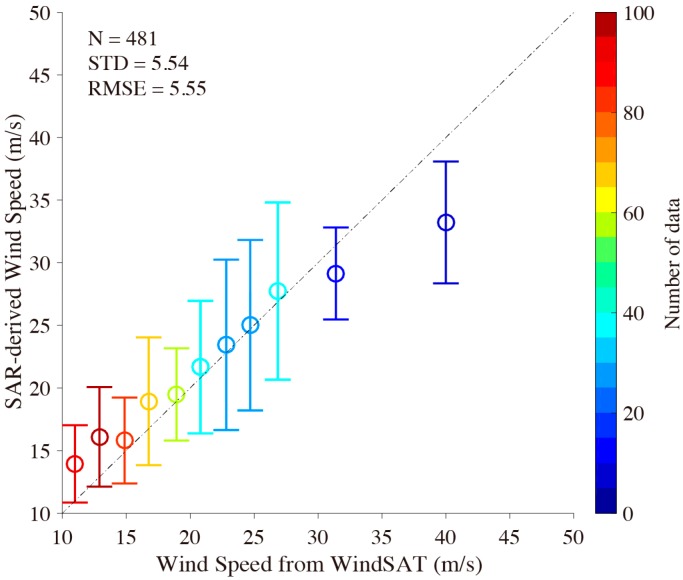
Comparison between SAR-derived wind speeds and measurements from Windsat grid winds between 10 m/s and 40 m/s for a 2 m/s bin, in which error bar represents the standard deviation (STD) of wind speed at each bin.

A comparison between SAR-derived wind speeds and measurements from Windsat grids data is shown in Figure 9. It is shown that there is around a 5.5 m/s difference in wind speed RMSE between the retrieval results from VH-polarization GF-3 SAR images and Windsat grids wind speeds and between 10 m/s and 40 m/s for a 2 m/s bin, in which the error bar represents the standard deviation (STD) of wind speed at each bin. As mentioned in Section 2, the “de-noised” procedure is not applied for the VH-polarization GF-3 SAR image in this study. We consider this probably explains why the analyzed result is worse than the 4.5 m/s RMSE of wind speeds in [23] when comparing retrieved wind speed in hurricanes from VH-polarization R-2 SAR images with Hurricane Research Division (HRD) wind vectors of the National Oceanic and Atmospheric Administration (NOAA). However, the validation still shows that VH-polarization GF-3 SAR has the capability to derive high wind speeds from typhoons, due to the fact that cross-polarization does not saturate as easily as the co-polarization signal.

## 5. Discussion

**Figure 10:**
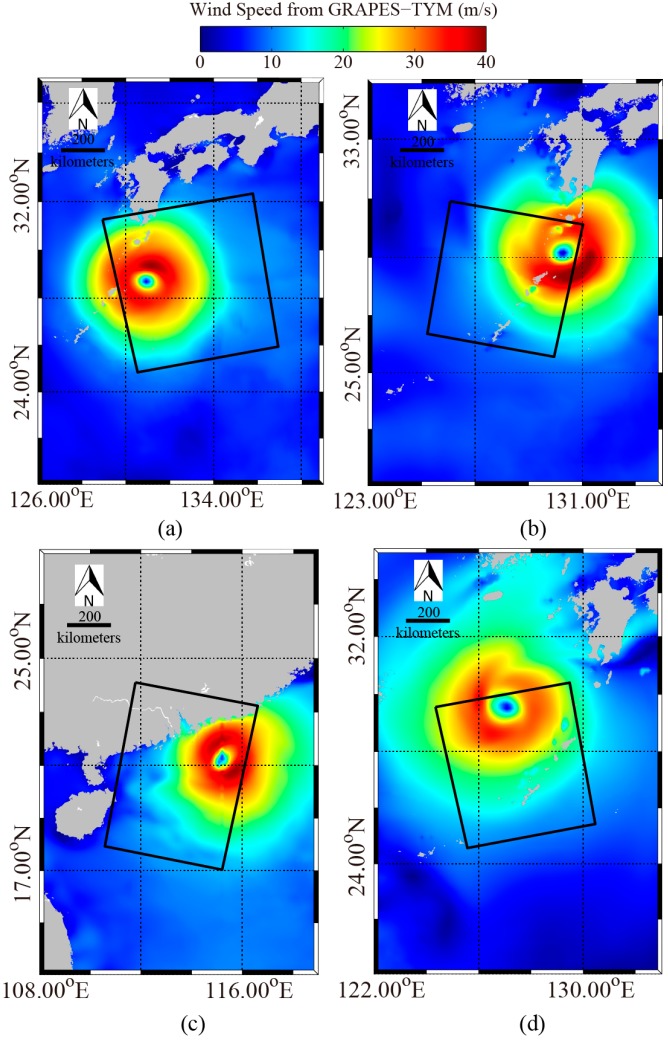
GRAPES-TYM wind maps, in which the rectangles represent the coverage of the corresponding three VH-polarization GF-3 SAR images in Figure 3. (**a**) GRAPES-TYM wind maps of typhoon e on 4 August 2017 at 09:00 UTC. (**b**) GRAPES-TYM wind maps of typhoon Noru on 4 August 2017 at 21:00 UTC. (**c**) GRAPES-TYM wind maps of typhoon Hato on 22 August 2017 at 22:00 UTC. (**d**) GRAPES-TYM wind maps of typhoon Talim on 16 September 2017 at 09:00 UTC.

Because there are not many matchups at wind speeds greater than 30 m/s, as shown in Figure 9, we also compared the SAR-derived winds with those from the 0.12 × 0.12° grids GRAPES-TYM model through the four GF-3 VH-polarization SAR images shown in Figure 3 in order to further investigate the accuracy for strong wind retrieval. The GRAPES-TYM wind maps are presented in Figure 10, in which the rectangles represent the spatial coverage of the four VH-polarization GF-3 SAR images.

**Figure 11:**
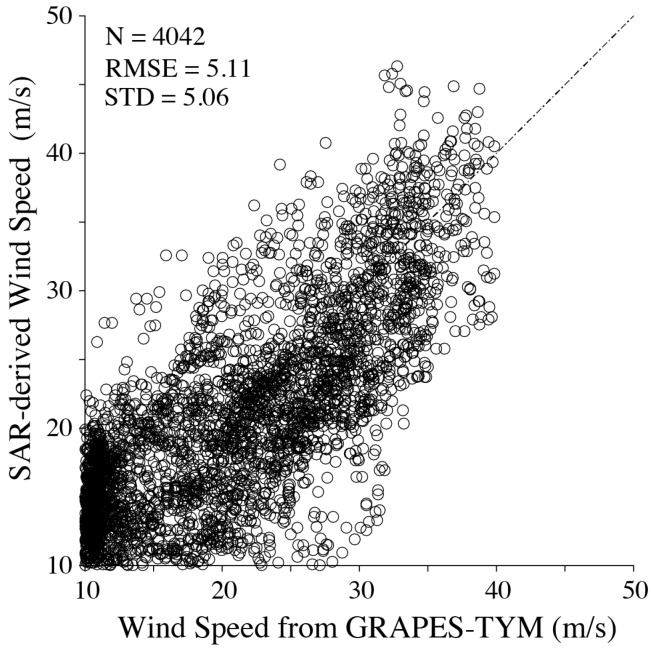
Comparison between SAR-derived wind speeds using the algorithm herein and GRAPES-TYM winds.

The comparison between SAR-derived winds and GRAPES-TYM winds is shown in Figure 11. It is found that the RMSE of wind speed is 5.1 m/s, which is less than the statistical analysis exhibited in Figure 8. This is not surprising as unique wind data were used for tuning and validating the proposed empirical algorithm. However, it is an obvious conclusion that the proposed algorithm works for typhoon winds retrieval from VH-polarization GF-3 SAR images without encountering the signal saturation problem when the wind speed is up to 40 m/s. Therefore, cross-polarization GF-3 SAR is an effective method for monitoring strong winds in typhoon conditions.

**Figure 12:**
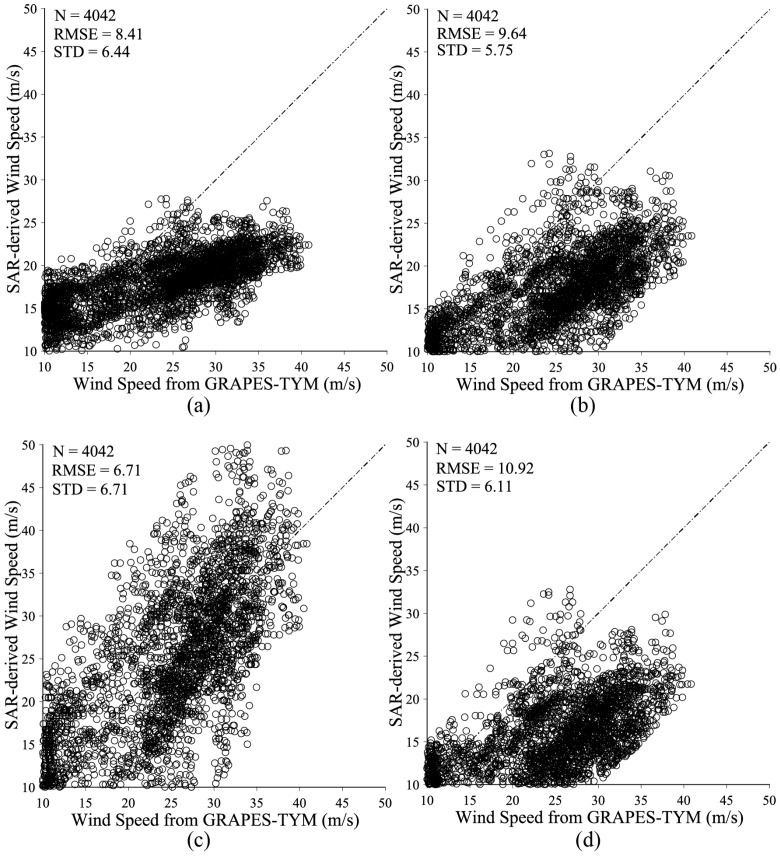
Comparison between SAR-derived wind speeds using the existing three algorithms and GRAPES-TYM winds. (**a**) using the algorithm proposed in [20]. (**b**) using the algorithm proposed in [22]. (**c**) using the algorithm proposed in [23]. (**d**) using the algorithm proposed in [25].

We also compared the SAR-derived results with GRAPES-TYM winds using the existing four cross-polarization algorithms proposed in [20,22,23,25]. Most of these have the same basic formulation as Equation (3) and are tuned through quad-polarization or dual-polarization R-2 SAR data. Although the algorithm proposed in [25] includes both term of wind speed and incidence angle, the NRCS has a linear relationship of the first order with the incidence angle. Figure 12 shows that the RMSE of wind speed is 6.4, 9.6, 6.7 and 10.9 m/s using the algorithms in [20,22,23,25], respectively. This analysis shows that these algorithms all perform less well than the results achieved using the proposed algorithm for noisy GF-3 SAR data.

## 6. Conclusions and Summary

To date, several co-polarization GMFs at C-band have been developed [3,4,5,6,7], which describe an empirical relationship between SAR NRCS and wind vector. These co-polarization GMFs have been successfully applied for various C-band SAR data, i.e., Envisat-ASAR [10], R-1/2 SAR [11,21], S-1 SAR [12] and GF-3 SAR [39,40]. However, the SAR-measured NRCS encounters a saturation problem under strong wind conditions [17,18,38], causing a limitation of the co-polarization GMF applicability. Recent achievements of R-2 signatures in cross-polarization reveal that the NRCS of cross-polarization SAR has a strong linear relationship with wind speed and it was found that the saturation boundary reaches 55 m/s [18]. The results proposed in [26] show that S-1 SAR in cross-polarization is also useful for wind monitoring.

In our study, we have investigated the dependence of wind speed and of radar incidence angle on NRCS of VH-polarization GF-3 SAR and then tuned an empirical wind retrieval algorithm. Seven GF-3 SAR images in VH-polarization were collected from the China Sea, and they were acquired in four typhoons during the summer of 2017. All images were processed as L-1B products, in which discontinuities still exist near the edge of different signature beams due to the instrumental noise. Among these images, there are three images collocated with GRAPES-TYM wind fields, which had good performance for the typhoon cases in the northwest Pacific Ocean and South China Sea in 2013 [35]. Analyzed results show that the NRCS linearly increases with wind speed without encountering the signal saturation problem for wind speeds up to 40 m/s. However, it was found that the NRCS of VH-polarization GF-3 SAR is not simply linearly related with radar incidence angle, which is probably caused by the different instrumental noise of each radar beam. Therefore, based on the recent achievements for strong wind retrieval C-band cross-polarization R-2 SAR [20] and S-1 SAR [26], an empirical algorithm has been tuned for wind retrieval for VH-polarization GF-3 SAR in typhoons through the collocated dataset, which takes into account the terms of wind speed and radar incidence angle.

The proposed empirical algorithm was applied for an additional four images in typhoons and the SAR-derived winds have been validated against Windsat winds at 0.25° grids. The comparison shows around 5.5 m/s RMSE of wind speed. We also compared the retrieved wind speed with GRAPES-TYM wind speeds, showing the RMSE of wind speed to be 5.1 m/s. Although the accuracy of wind retrieval for VH-polarization GF-3 SAR is greater than that (around 2 m/s STD) for co-polarization C-band SAR using the traditional methodology of GMF, the advantage of the proposed empirical algorithm is that it is able to operate without prior knowledge of wind direction and works under strong wind conditions without encountering the signal saturation problem. It is necessary to establish that the proposed algorithm only works for wind speeds greater than 10 m/s and homogeneous sub-scenes, as the SAR signature is weak in the presence of noise and rainfall in typhoons.

In summary, we conclude that the proposed algorithm is a promising potential technique for wind retrieval from C-band VH-polarization GF-3 SAR image in typhoons. Increasingly, more GF-3 SAR images will be captured in extreme winds by our institute and the National Satellite Ocean Application Service (NSOAS). In particular, the dependence of instrumental noise on NRCS from VH-polarization GF-3 SAR acquired in GLO and WSW mode can be investigated. In future, we could further tune the empirical algorithm in order to improve the accuracy of retrieved winds from VH-polarization GF-3 SAR images. 

## Figures and Tables

**Table 1:** Coefficients in Equations (3)–(5), which are determined from the collocated data in our study.

A	0.152	B	−28.378			
θ	10°~20°	20°~25°	25°~30°	30°~35°	35°~40°	40°~50°
α	−0.016	−0.025	−0.013	−0.004	−0.031	−0.009
β	0.023	−0.164	0.198	0.359	−0.193	0.012
C_1_	0.015	−0.001	−0.080	−0.046	0.198	0.067
C_2_	−0.400	−0.360	4.264	2.972	−15.139	−5.675
C_3_	−22.428	−16.133	−81.322	−73.816	261.555	93.644

## Appendix A

**Table A1:** Information of seven VH-polarization GF-3 SAR images and corresponding typhoons.

Typhoon	Acquisition Time (YYYY-MM-DD)	Cyclone Eye (° E, ° N)	Maximum Wind Speed (m/s)	Radius of Maximum Wind Speed (km)	Central Pressure (hPa)
Noru	2017-08-02 21:09	135.6, 26.1	45	50	950
Noru	2017-08-04 09:12	131.4, 28.4	40	100	960
Noru	2017-08-04 21:26	130.7, 29.0	40	80	960
Hato	2017-08-22 22:23	117.1, 20.8	34	80	970
Doksuri	2017-09-13 22:14	114.3, 15.8	23	180	990
Talim	2017-09-14 21:29	124.3, 27.4	50	80	940
Talim	2017-09-16 09:30	126.1, 29.4	38	40	965
